# Congregational Informal Support Networks for Older Adults Without Advocates in Black Churches

**DOI:** 10.1093/geroni/igaf122.2985

**Published:** 2025-12-31

**Authors:** Miriam Gofine, Carolyn Berry, Stephanie Zhang, Antoinette Schoenthaler

**Affiliations:** New York University, Brookline, Massachusetts, United States; New York University, New York, New York, United States; New York University, New York, New York, United States; New York University, New York, New York, United States

## Abstract

Increasing proportions of older adults (OAs) have unmet care needs due to limited or no familial support (“Older Adults Without Advocates” [OAWA] or “elder orphans”). This prevalence is expected to rise, especially among Black/African American (B/AA) OAs. Both the peer-reviewed and grey literatures suggest that many OAWA receive at least some support from church-based informal (i.e., unpaid) social networks. To our knowledge, no published research exists on B/AA OAWA, but prior work emphasizes the significance of spiritual, emotional, and instrumental support provided by volunteers in congregational informal support networks (CISN) to community-dwelling B/AA OAs more generally. This study synthesizes the work of expert scholars on church-based informal social support networks of B/AA OAs with the Convoy of Care Model and the American Geriatrics Society’s identification of “elder orphans” as a distinct clinical population to explore the role of CISN in B/AA OAWA’s care networks. Guided by a community-engaged framework, 18 semi-structured interviews with OAWA and CISN members affiliated with 12 predominantly B/AA Baptist and Methodist churches across New York City were thematically analyzed. Preliminary results recognize CISN as a critical element of care networks of OAs and OAWA alike, identifying discordance between clinical and social definitions of OAWA and noting overlap between OAWA and CISN groups. This research enhances understanding of caregiving patterns related to OAWA within urban Black/African American faith communities, highlighting the essential role of CISN in promoting successful aging in place for urban B/AA OAWA.

